# Supplementary material for: Mitigating ion flux vortex enables reversible zinc electrodeposition
Source: Nat Commun. 2025 Aug 8;16:7312. doi: 10.1038/s41467-025-62470-x (PMC12331982; doi:10.1038/s41467-025-62470-x)
Supplement: Supplementary file 1 — Supplementary Information [file 41467_2025_62470_MOESM1_ESM.pdf]

Supplementary Information

Mitigating ion flux vortex enables reversible zinc electrodeposition

Yuhang Dai[1,2,#], Wenjia Du[2,#], Haobo Dong[1,3,#], Xuan Gao[1,2,*], Chang Su[4], Partha P. Paul[5,6], Bratislav Lukic[5,6], Chengyi Zhang[7], Chumei Ye[8], Jinghao Li[9], Wei Zong[2], Jianwei Li[1], Yiyang Liu[1], Alexander Rack[6], Liqiang Mai[9,*], Paul R. Shearing[2,*], Guanjie He[1,*]

[1] Christopher Ingold Laboratory, Department of Chemistry, University College London, London WC1H 0AJ, UK

[2] Department of Engineering Science, University of Oxford, Oxford OX1 3PJ, UK

[3] School of Future Technology, South China University of Technology, Guangzhou 510641, China

[4] Nuffield College, University of Oxford, Oxford OX1 1NF, UK

[5] Henry Royce Institute, Department of Materials, The University of Manchester, Manchester M13 9PL, UK

[6] ESRF - The European Synchrotron, 71 Avenue des Martyrs, Grenoble 38000, France

[7] School of Chemical Sciences, University of Auckland, Auckland 1010, New Zealand

[8] Department of Materials Science and Metallurgy, University of Cambridge, Cambridge CB3 0FS, UK

[9] State Key Laboratory of Advanced Technology for Materials Synthesis and Processing, Wuhan University of Technology, Wuhan 430070, China

[#] These authors contributed equally to this work

[*] Email: xuan.gao@eng.ox.ac.uk; mlq518@whut.edu.cn; paul.shearing@eng.ox.ac.uk; g.he@ucl.ac.uk.

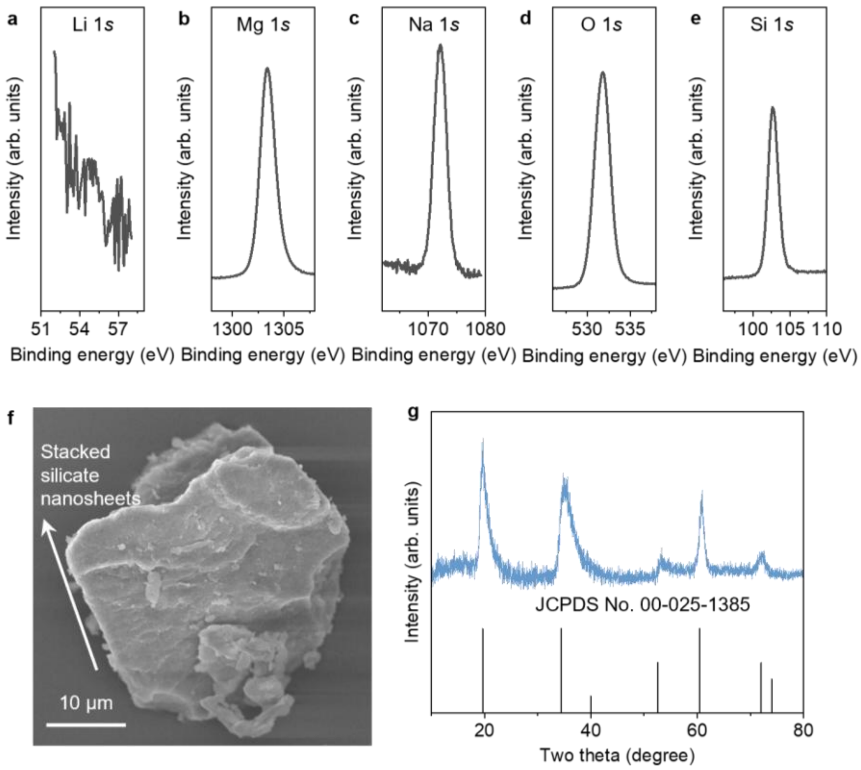

Supplementary Figure 1. Characterizations of LAPONITE powder. a-e, X-ray photoelectron spectroscopy (XPS) spectra. **f,** Scanning electron microscope (SEM) image. **g,** X-ray diffraction (XRD) pattern. The JCPDS No. 00-025-1385 corresponds to $Na_{0.2}(Mg, Li)_3Si_4O_{10}(OH)_2 \cdot 4H_2O$.

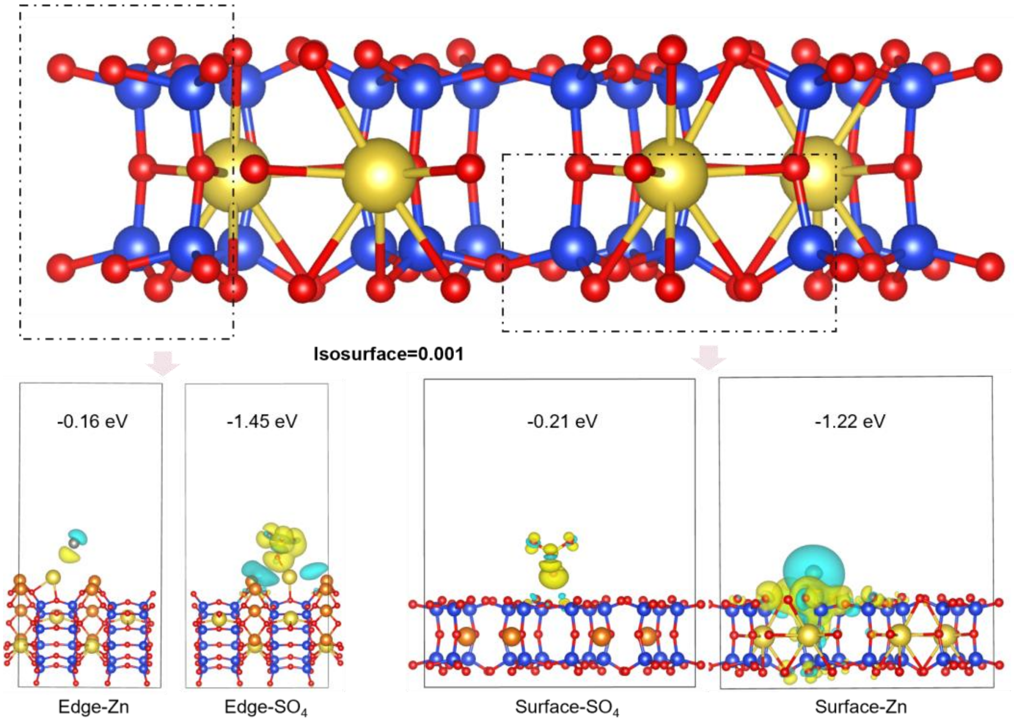

Supplementary Figure 2. Density functional theory (DFT) calculation result of LAPONITE lamella. Analysis of charge density difference to investigate the adsorption behavior of Zn^{2+} and SO_4^{2-} on distinct sites (edge or surface) of LAPONITE. The chain structure of LAPONITE consists of [SiO_4] tetrahedra with Mg atoms between the chains. The blue area indicates electron loss, while the yellow area presents electron enrichment. The adsorption energy to each ion (cation) is labeled in the figure.

The primary source of the significant differential charge density difference between Zn^{2+} and SO_4^{2-} at the surface is their distinct interactions with oxygen atoms. Specifically, zinc close to the surface coordinates with the surface oxygen to form bonds with an adsorption energy of -1.22 eV, while the oxygen in the sulfate group cannot bond tightly with the surface oxygen, resulting in a lower adsorption energy of -0.21 eV. At the edge, the oxygen is already saturated by interlayer metal atoms, making the exposed metal atoms more likely to coordinate with the sulfate group (-1.45 eV) rather than zinc (-0.16 eV). In addition, the formation energy when zinc enter the LAPONITE structures is calculated to be -1.73 eV, indicating a stable configuration that promotes long-cycle stability of the battery.

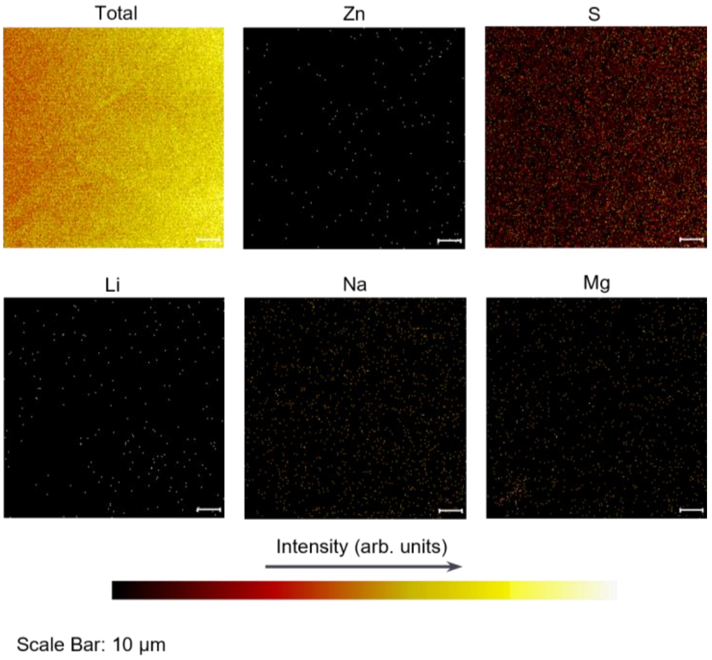

Supplementary Figure 3. Elemental mapping of the LAPONITE surface from time-of-flight secondary ion mass spectrometry (TOF-SIMS) test.

After one cycle of deposition, LAPONITE was peeled off from the LAPO@Zn anode. The TOF-SIMS result was collected from negative mode. The Zn signal is low, while the S signal is high, indicating that LAPONITE selectively adsorbs SO_4^{2-} while allowing Zn^{2+} to pass through.

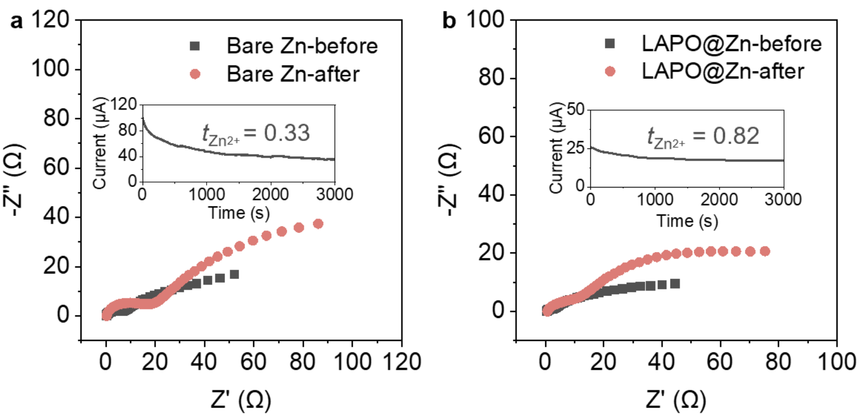

Supplementary Figure 4. Impedance spectra of bare Zn and LAPO@Zn electrodes before and after polarization. a, Electrochemical impedance spectroscopy (EIS) spectra based on bare Zn electrodes. **b,** EIS spectra based on LAPO@Zn electrodes. The inset shows the variation of current with time during polarization at an applied voltage of 10 mV and room temperature.

The transference number (t_{Zn2+}) values were measured by the potentiostatic polarization method, and determined by the *Evans* equation as below:

$$t_{Zn}^{2+} = \frac{I_S(\Delta V - I_0 R_0)}{I_0(\Delta V - I_S R_S)} \quad (1)$$

where I_0 and I_s represent the currents at the initial and steady states, respectively. R_0 and R_s represent the interfacial resistances prior to and following polarization, respectively. The t_{Zn2+} values of bare Zn and LAPO@Zn are 0.33 and 0.82, respectively.

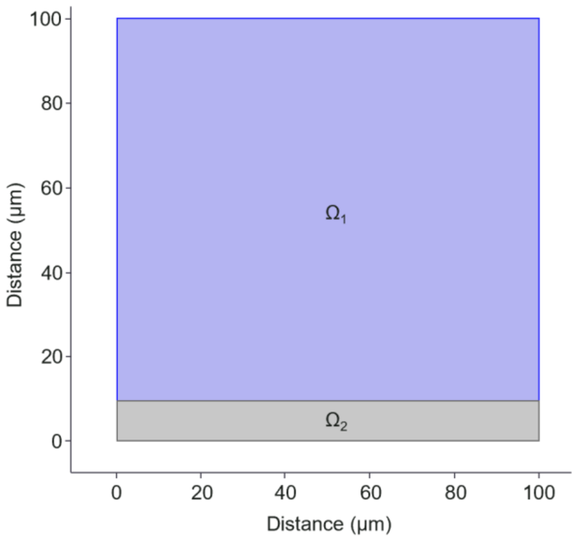

Supplementary Figure 5. Calculation model using the computational fluid dynamics (CFD) method for ion transport upon nanochannels.

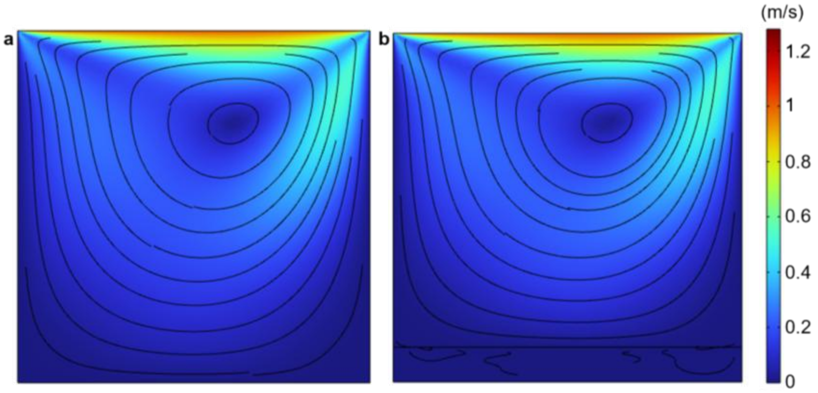

Supplementary Figure 6. Ionic concentration fields upon bare Zn and coating. a, Ionic concentration fields upon bare Zn. **b,** Ionic concentration fields upon coating.

In a 10 μm×10 μm two-dimensional region on the electrode surface, an artificial vortex (top-driven flow) was generated to simulate the smallest-scale turbulent motion of the solution at the electrode interface. Fluid motion within the porous coating adheres to Darcy's law, as depicted in the velocity field. The convection-diffusion equation is employed to depict the movement of Zn^{2+} and SO_4^{2-} concentrations near the electrode surface. The diffusion rate of ions within the LAPONITE coating is influenced by the concentration of absorbed SO_4^{2-}.

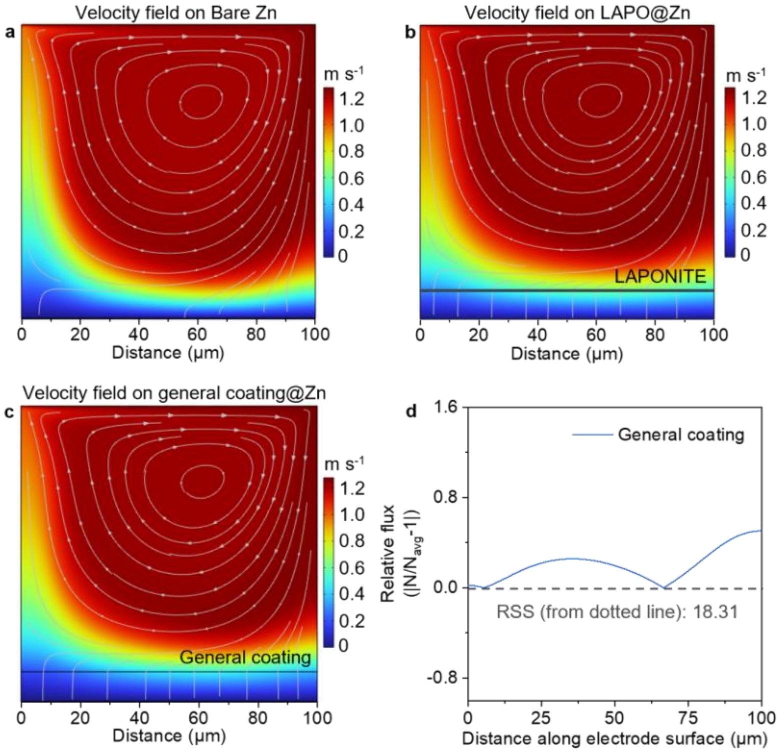

Supplementary Figure 7. Velocity fields on different substrates. a-c, Ionic concentration fields upon bare Zn (**a**), LAPO@Zn (**b**), and general coating@Zn (**c**). **d,** Zn^{2+} flux density on the general coating@Zn surface.

N_{local} and $N_{average}$ represent local and average Zn^{2+} flux densities, respectively. Deviation represents the residual sum of squares of individual data points relative to the dotted line. The general coating (like a conventional nanopore channeled coating) restricts ion diffusion, resulting in a more uniform distribution of metal ion concentration compared to bare Zn but less uniform than achieved with LAPO@Zn.

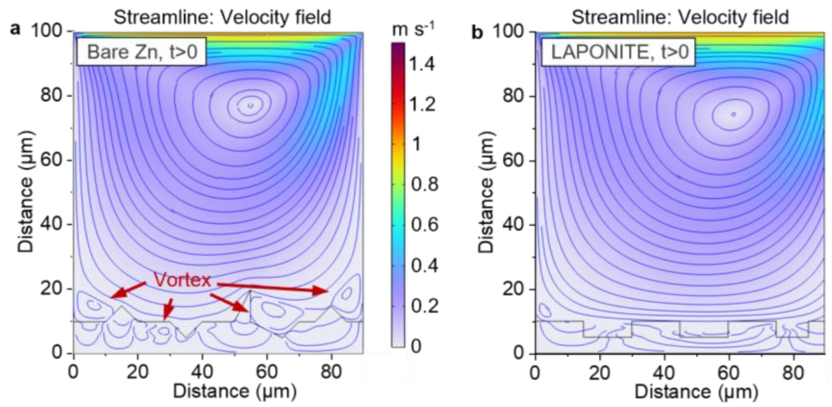

Supplementary Figure 8. Velocity fields upon cycled electrodes. a, Velocity field on bare Zn. **b,** Velocity field on LAPO@Zn.

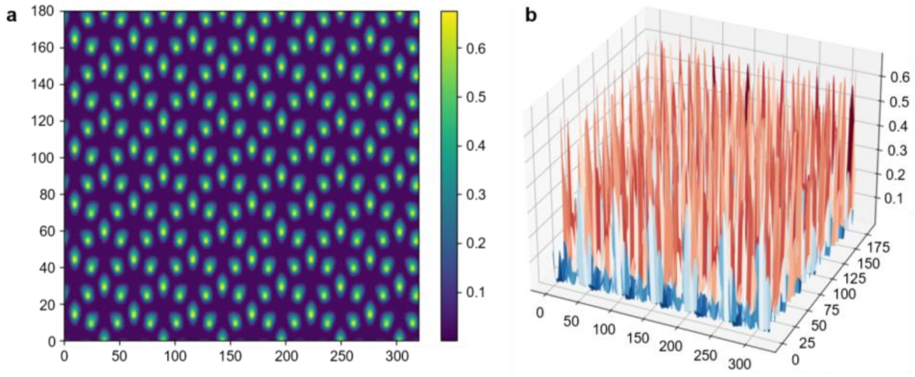

Supplementary Figure 9. Electron distribution simulation. a, Visualization of the electron localization function. **b,** Related potential of the surface of LAPONITE.

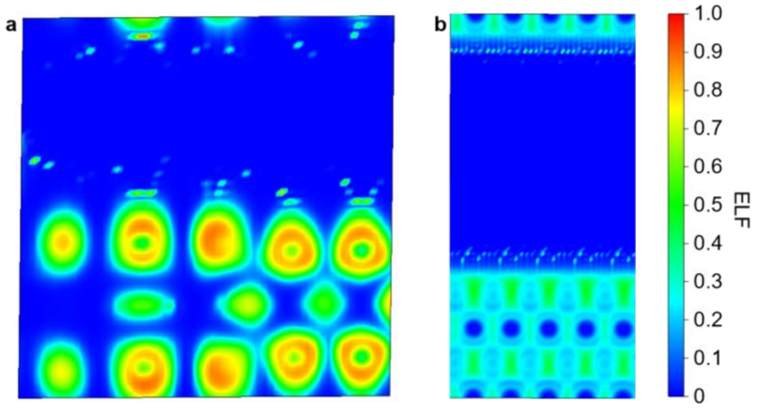

Supplementary Figure 10. DFT calculation of the electron localization function (ELF). a, On LAPONITE slabs. **b,** On Zn slabs.

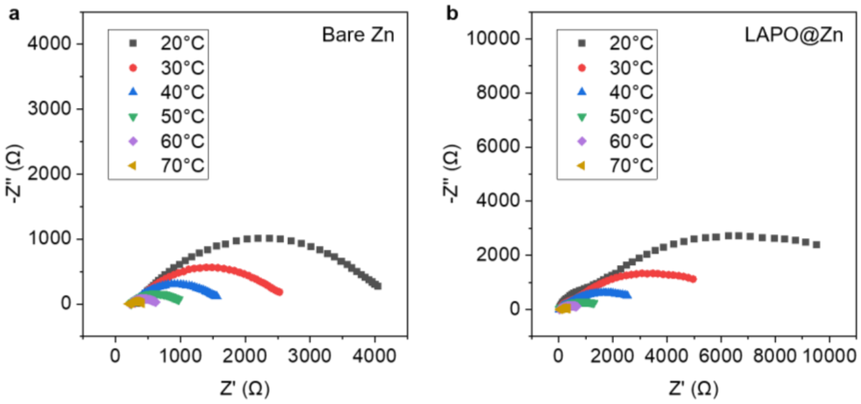

Supplementary Figure 11. EIS spectra at different temperatures. a, Bare Zn symmetric cells. **b,** LAPO@Zn symmetric cells.

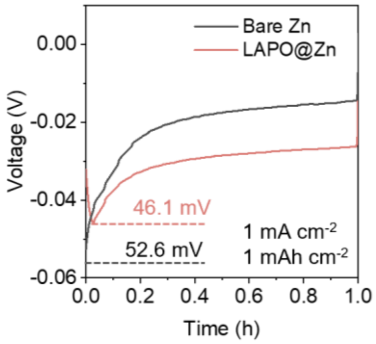

Supplementary Figure 12. First-cycle discharge profiles of Zn||Zn symmetric cells using bare Zn and LAPO@Zn electrodes.

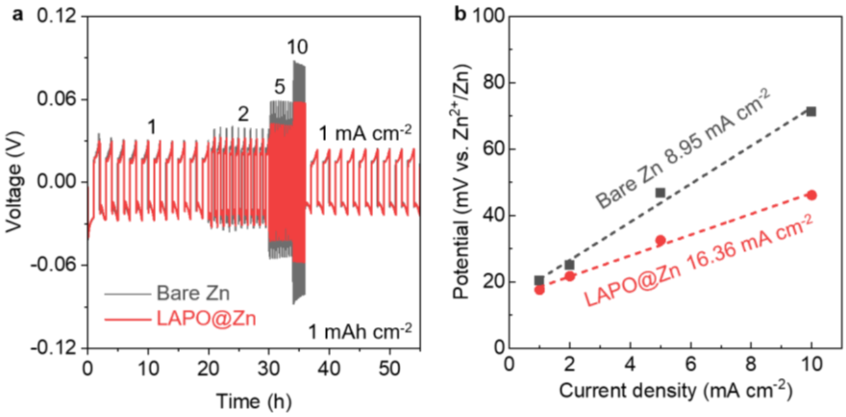

Supplementary Figure 13. Rate performance based on different electrodes. a, Plating/stripping curves of bare Zn and LAPO@Zn symmetric cells at 1 mA cm^{-2}, 2 mA cm^{-2}, 5 mA cm^{-2}, 10 mA cm^{-2} with a fixed capacity of 1 mAh cm^{-2}. **b,** Exchange current density calculated from rate performance of Zn||Zn symmetric cells in (**a**).

This analysis allowed us to determine the exchange current densities following the equation provided below:

$$i = i_0 \frac{F}{RT}\frac{\eta}{2} \qquad (2)$$

where i is the current density, i_0 is the exchange current density, F is the Faraday constant, R is the gas constant, T is the absolute temperature, and η is the overpotential.

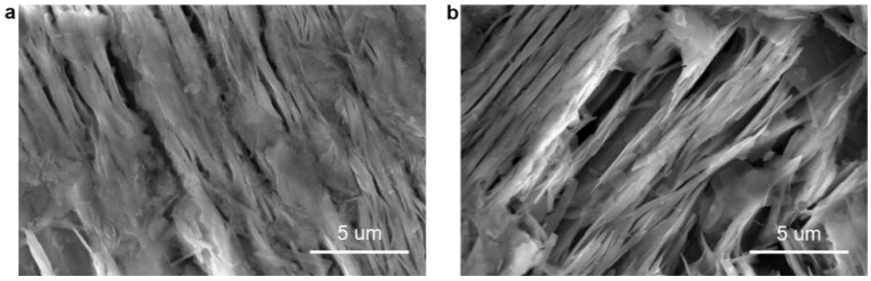

Supplementary Figure 14. Zn morphology beneath the LAPONITE coating. a,b, SEM images of the LAPO@Zn electrodes after 1 h plating at 1 mA cm^{-2}. The images were collected after removing the surface LAPONITE layer.

The exposed Zn surface displays compact, vertically aligned grains, directly confirming the guided growth along the (100) plane. This orientation is favourable for suppressing dendrites and enhancing cycling reversibility.

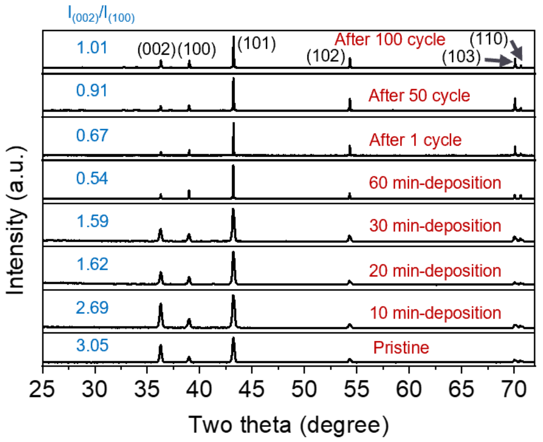

Supplementary Figure 15. GIXRD patterns of LAPO@Zn at different stages, including pristine condition (also the same to the original Zn foil), after 10 min, 20 min, 30 min, and 60 min deposition at 1 mA cm^{-2}, as well as after 1, 50, and 100 cycles of deposition/stripping cycles, respectively.

The $I_{(002)}/I_{(100)}$ ratio decreases from 3.05 (pristine) to 2.69, 1.62, 1.59, and 0.54 after 10, 20, 30, and 60 min of deposition at 1 mA cm^{-2}, indicating a gradual enhancement of the (100) orientation guided by the LAPONITE coating. After 1, 50, and 100 cycles, the ratio gradually increases to 0.67, 0.91, and 1.01, respectively, likely due to weakened orientation control caused by interfacial degradation from HER and the accumulation of zinc hydroxide sulfate (ZHS) byproducts.

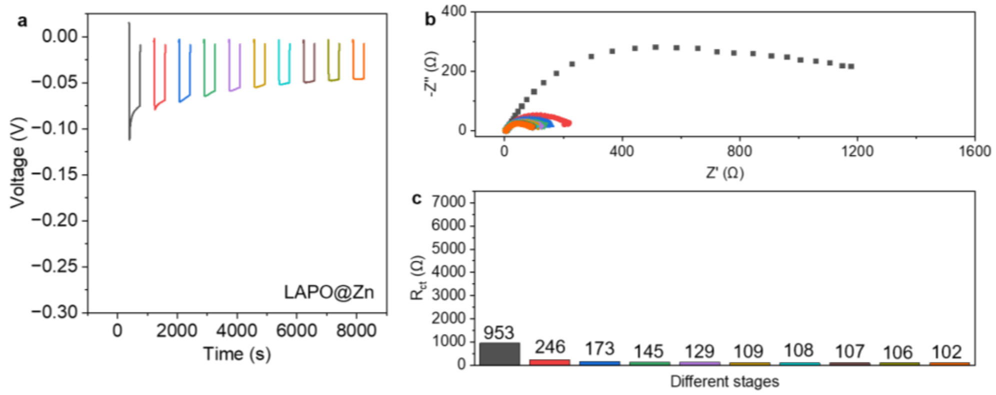

Supplementary Figure 16. Operando EIS analysis of LAPO@Zn symmetric cells under stepwise Zn deposition at 1 mA cm^{-2}. a, Galvanostatic discharge curve with 6-min discharge intervals. **b**, Nyquist plots collected at the start of each segment. **c**, Evolution of charge transfer resistance (R_{ct}), showing rapid drop and stabilization from the fourth stage onward.

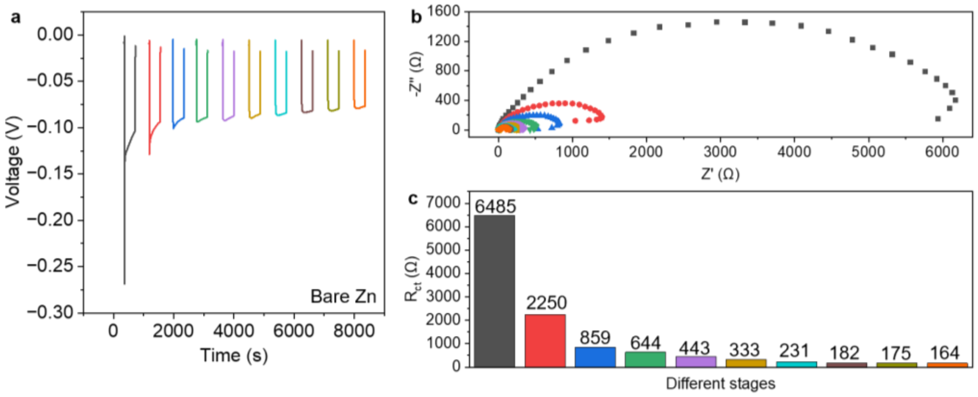

Supplementary Figure 17. Operando EIS analysis of bare Zn symmetric cells under stepwise Zn deposition at 1 mA cm^{-2}. a, Galvanostatic discharge curve with 6-min discharge intervals. **b**, Nyquist plots collected at the start of each segment. **c**, Evolution of R_{ct}, showing rapid drop and stabilization from the fourth stage onward.

The operando EIS results in Supplementary Figs. 16 and 17 reveal distinct interfacial dynamics between LAPO@Zn and bare Zn. LAPO@Zn exhibits a rapid drop in R_{ct} to 246 Ω, followed by stabilization below 110 Ω after the fourth segment, indicating fast interface stabilization. In contrast, bare Zn starts with 2250 Ω and shows a much slower decline, only reaching 231 Ω by the seventh stage, with further decline afterward.

These results suggest that LAPONITE rapidly suppresses interfacial ion flux vortices and facilitates homogeneous Zn^{2+} transport, leading to steady deposition. The bare Zn interface, lacking this regulation, experiences persistent impedance and delayed stabilization. This operando validation strongly supports our proposed vortex suppression mechanism.

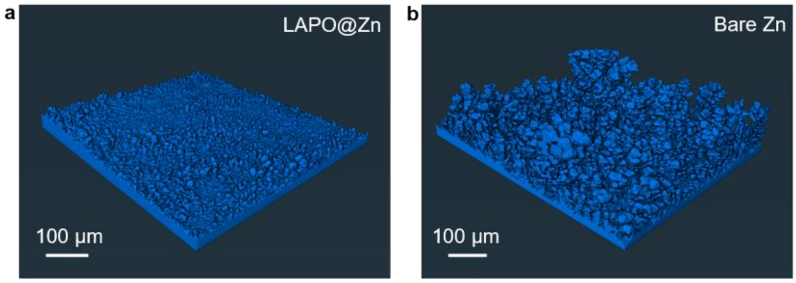

Supplementary Figure 18. 3D rendered Zn images after in-situ X-CT testing of LAPO@Zn (**a**) and Bare Zn (**b**).

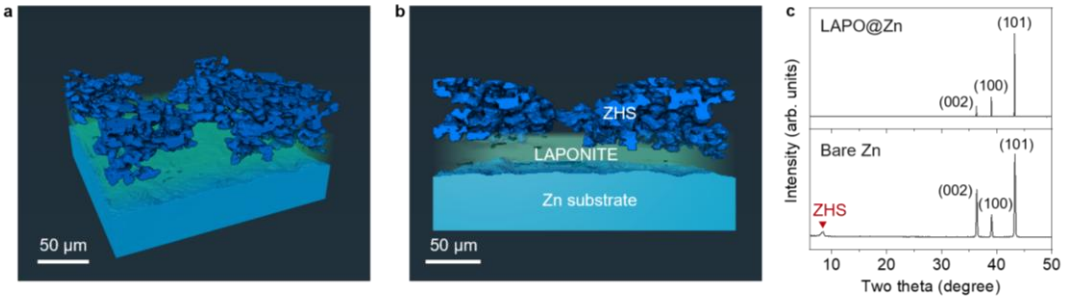

Supplementary Figure 19. ZHS detection from in-situ X-ray CT investigation and XRD pattern. a,b, Overlapped segmentation images of LAPO@Zn after in-situ X-CT testing. **c,** GIXRD patterns of bare Zn and LAPO@Zn after cycling.

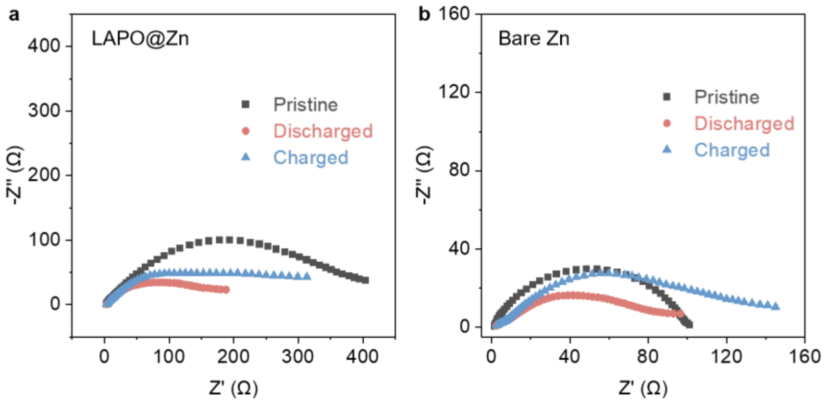

Supplementary Figure 20. In-situ EIS spectra. These spectra were recorded at pristine, discharged, and charged states during the first cycle, including LAPO@Zn (**a**) and bare Zn (**b**) symmetric cells.

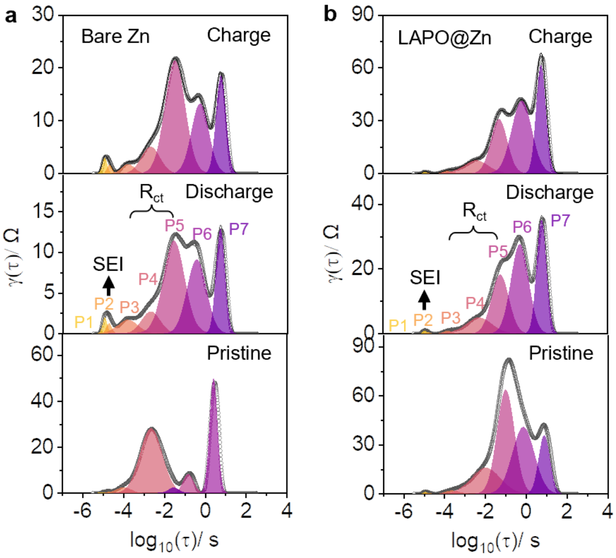

Supplementary Figure 21. The distribution of relaxation times (DRT) plot of the in-situ EIS data. These spectra attributed to bare Zn (**a**) and LAPO@Zn (**b**), after cycling. The P3-P5 peaks are attributed to the R_{ct}. (Specific parameters can be found in Supplementary Table 2).

By conducting DRT analysis towards the *in-situ* EIS spectra, we partitioned the time constant into seven segments, where P2 corresponds to the solid-electrolyte interphase (SEI), and P3-P5 corresponds to the interfacial R_{ct}. For the bare Zn electrode, a distinct P2 peak appeared after 1 h of discharging at 1 mA cm^{-2}, which remained after 1 h of charging at 1 mA cm^{-2}, indicating the irreversible formation of ZHS-contained SEI. In contrast, in the LAPO@Zn system, the signal of P2 peak is weak after one discharge and disappears when charging back, suggesting that the amount of ZHS generated on the LAPO@Zn surface is sparse and reversible. Consequently, the variation in the R_{ct} of the LAPO@Zn surface is also remarkably small due to the significant suppression of surface byproducts, whereas the R_{ct} of the bare Zn becomes significant after cycling.

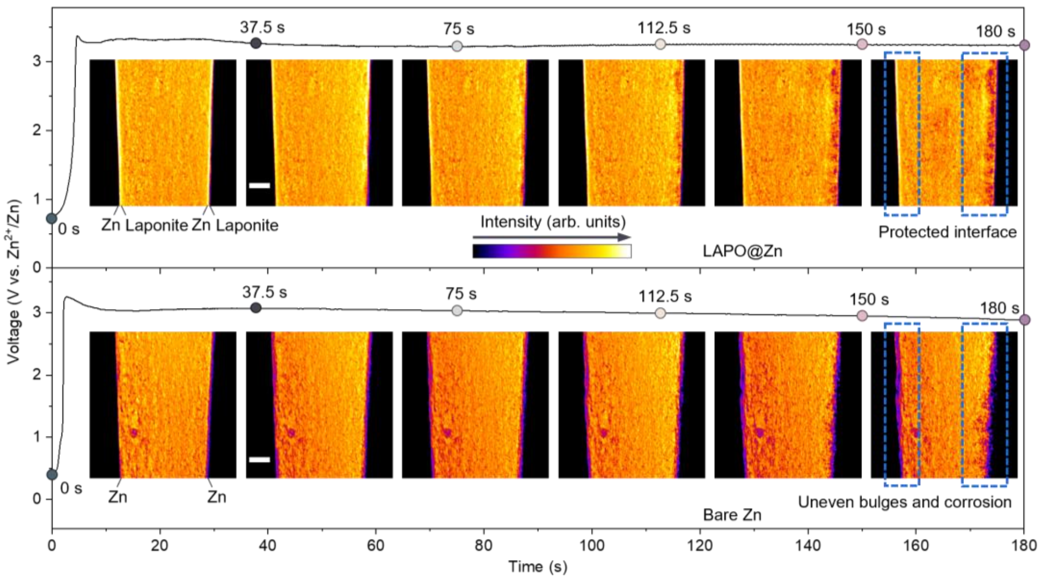

Supplementary Figure 22. Operando X-ray radiography of symmetrical cells with bare Zn and LAPO@Zn electrodes. The scale bar in the images represents 50 μm.

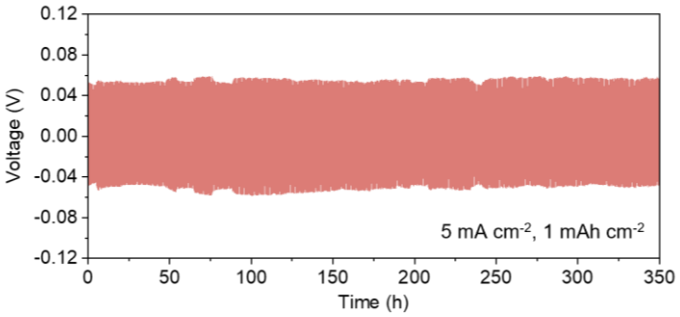

Supplementary Figure 23. Galvanostatic cycling curves of LAPO@Zn symmetric cells at 5 mA cm^{-2} with a fixed capacity of 1 mAh cm^{-2}.

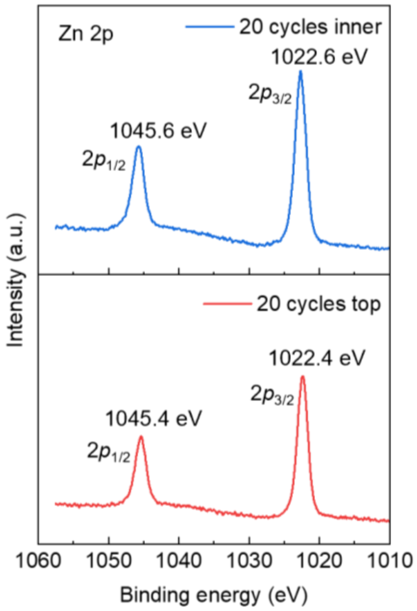

Supplementary Figure 24. XPS spectra of the LAPONITE+CMC interlayer after 20 deposition/stripping cycles at 1 mA cm^{-2} for 1 h. The red and blue curves correspond to the top surface and middle region of the coating, respectively.

Both spectra show Zn^{2+} signals with no evidence of metallic Zn (Zn^0, which typically exhibits a peak at around 1021.5 eV), indicating that Zn reduction occurs only after Zn^{2+} passes through the LAPONITE coating and reaches the conductive substrate.

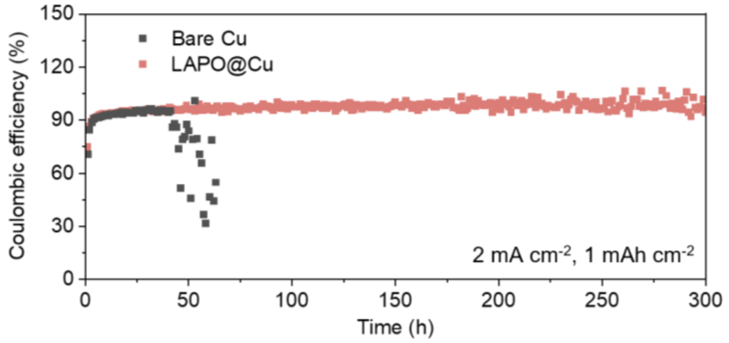

Supplementary Figure 25. Coulombic efficiency (CE) profile of Zn||Cu coin cells using bare Cu and LAPO@Cu electrodes at 2 mA cm^{-2}, with a fixed capacity of 1 mAh cm^{-2}.

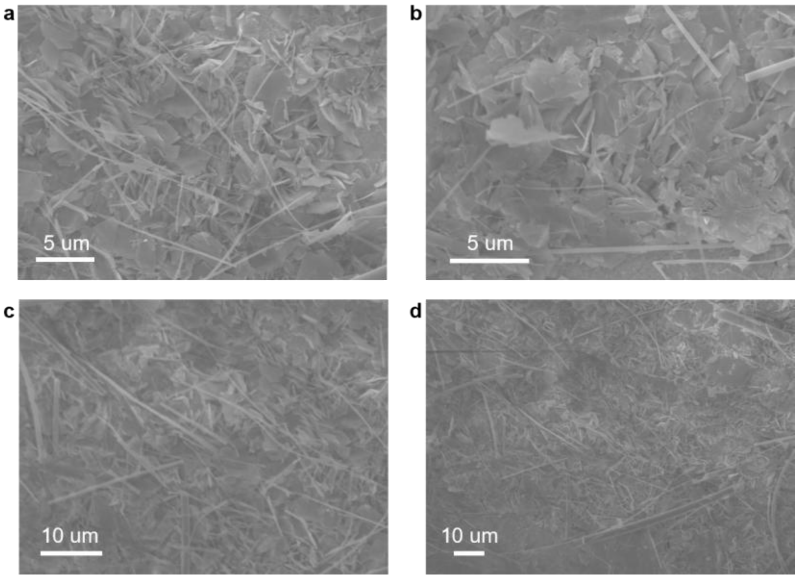

Supplementary Figure 26. SEM images of the Zn electrodes coated solely with CMC (CMC@Zn) foils after 1 h plating at 1 mA cm^{-2}.

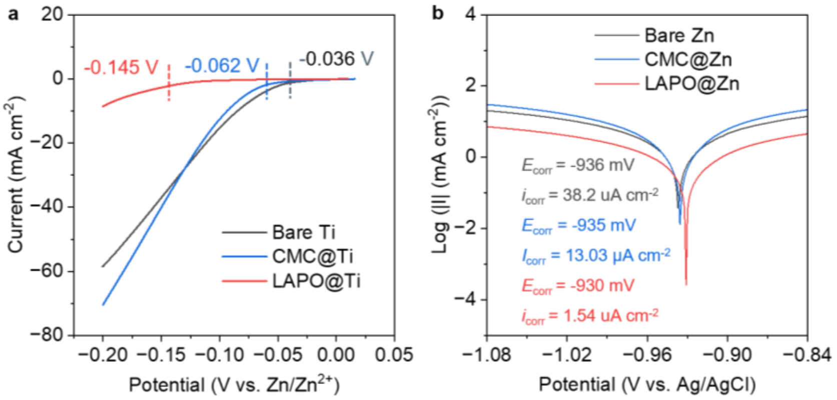

Supplementary Figure 27. a, Linear sweep voltammetry (LSV) curves of bare Ti, CMC@Ti, and LAPO@Ti electrodes. **b,** Linear polarization curves of bare Zn, CMC@Zn, and LAPO@Zn electrodes.

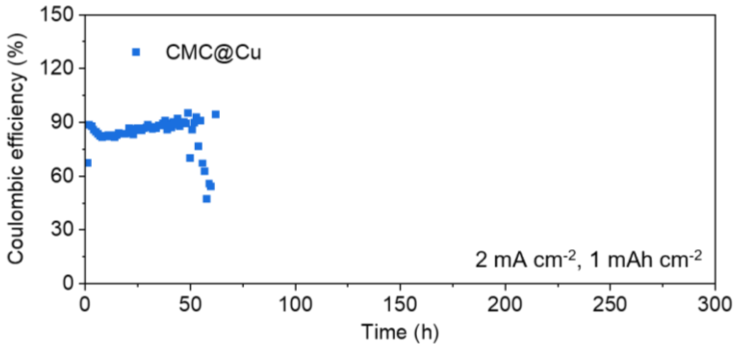

Supplementary Figure 28. Coulombic efficiency (CE) profile of Zn||Cu coin cells using CMC@Cu electrodes at 2 mA cm^{-2}, with a fixed capacity of 1 mAh cm^{-2}.

Supplementary Figs. 26-28 prepared further investigated solely CMC coated electrodes. To isolate the effect of CMC, we prepared CMC@Zn with the same loading (1 mg cm^{-2}) as the LAPONITE coating in LAPO@Zn. SEM images after Zn plating revealed disordered dendrite formation and detached SiO_2 fibers (Supplementary Fig. 26), similar to those observed on bare Zn (Fig. 3a in the main text), indicating limited regulation of Zn deposition. Furthermore, CMC@Zn exhibited higher HER activity (with a more positive onset potential) and corrosion current density compared to LAPO@Zn (Supplementary Fig. 27). In Zn||Cu cells, CMC@Cu failed within 50 h (Supplementary Fig. 28), whereas LAPO@Cu maintained stable cycling for over 200 h (Supplementary Fig. 25). These results confirm that the uniform deposition and improved reversibility mainly originate from the LAPONITE component, rather than CMC.

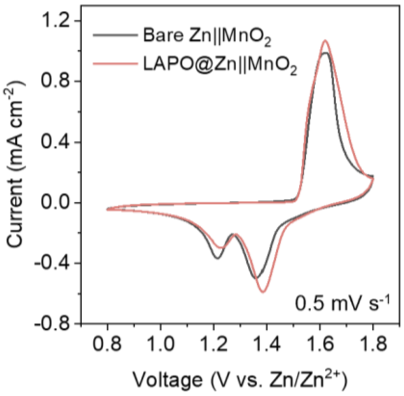

Supplementary Figure 29. Cyclic voltammetry (CV) curves of bare Zn||MnO_2 and LAPO@Zn||MnO_2 coin cells at a scan rate of 0.5 mV s^{-1}.

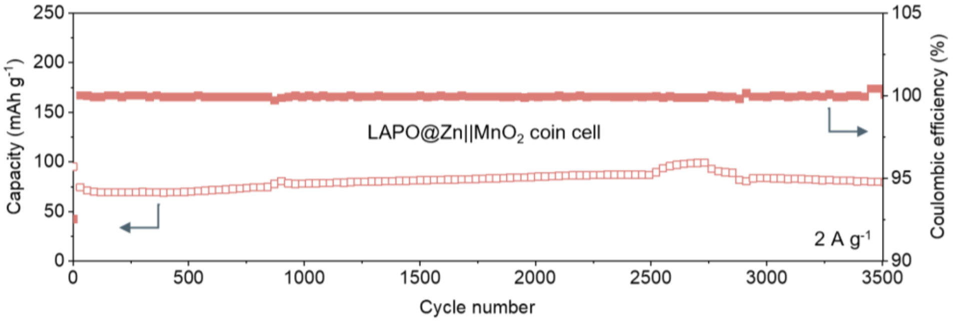

Supplementary Figure 30. Galvanostatic cycling performance of LAPO@Zn||MnO_2 coin cells at 2 A g^{-1}.

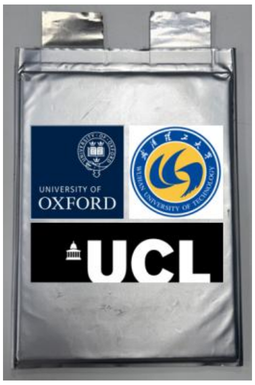

Supplementary Figure 31. Optical image of the assembled LAPO@Zn||MnO_2 pouch cell.

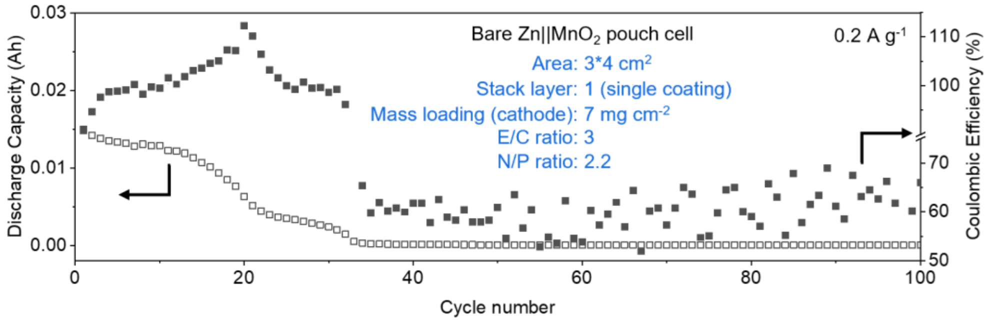

Supplementary Figure 32. Galvanostatic cycling performance of the bare Zn||MnO_2 pouch cell at 0.2 A g^{-1}.

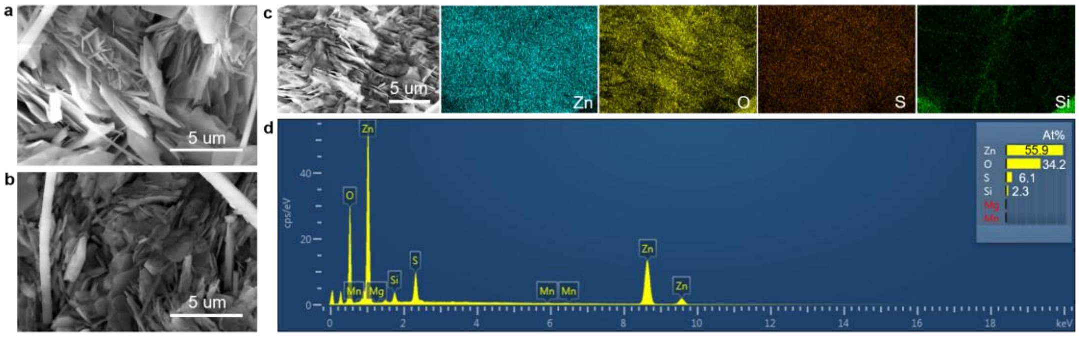

Supplementary Figure 33. Post-mortem characterizations of the bare Zn anode after 100 cycles in a Zn||MnO_2 pouch cell. **a,b,** SEM images. **c,** SEM image with corresponding energy dispersive spectrometer (EDS) mapping. **d,** Energy-dispersive X-ray (EDX) spectrum.

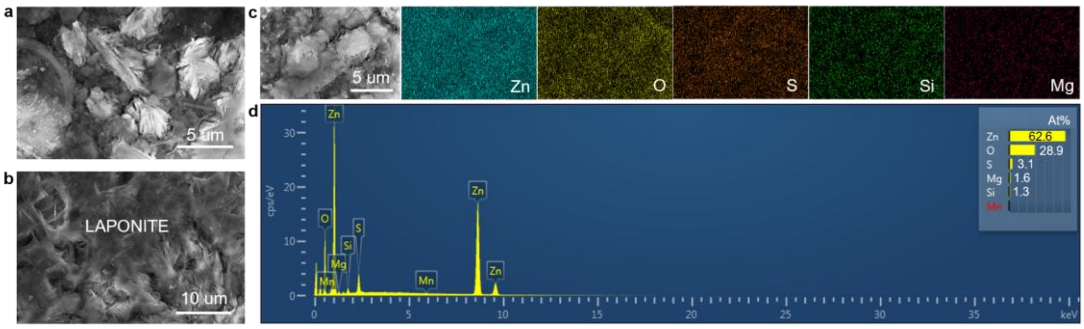

Supplementary Figure 34. Post-mortem characterizations of the LAPO@Zn anode after 100 cycles in a Zn||MnO_2 pouch cell. **a,b,** SEM images. **c,** SEM image with corresponding EDS mapping. **d,** EDX spectrum.

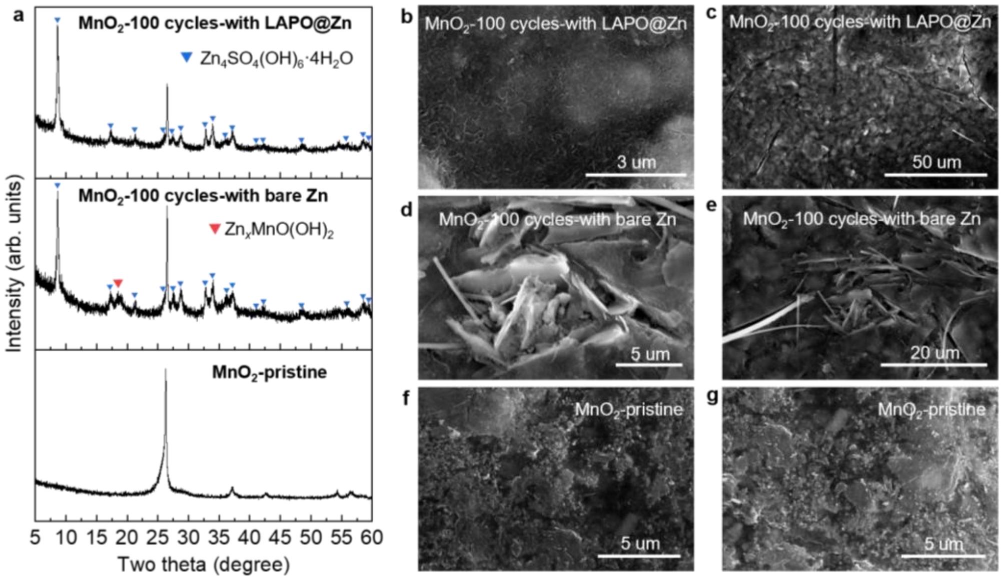

Supplementary Figure 35. Post-mortem characterizations of the MnO_2 cathodes after 100 cycles in bare Zn||MnO_2 and bare LAPO@Zn||MnO_2 pouch cell. **a,** XRD patterns of pristine MnO_2, cycled with bare and LAPO@Zn anodes. **b-g,** SEM images of MnO_2 cycled with LAPO@Zn anodes (b,c), bare Zn anodes (d,e) and pristine MnO_2.

Post-mortem analysis of the anodes and cathodes extracted from pouch cells after 100 cycles provides insight into the degradation mechanisms (Supplementary Figs. 33-35). The bare Zn anode exhibited severe damage, including randomly stacked dendrites and significant corrosion (Supplementary Fig. 33a-c), accompanied by elevated oxygen (34.2%) and sulfur (6.1%) levels, suggesting the formation of $Zn_4SO_4(OH)_6 \cdot nH_2O$ byproducts. A detectable silicon signal (2.3%) was also observed (Supplementary Fig. 33d), indicating mechanical damage to the glass fiber separator.

In contrast, the LAPO@Zn anode retained vertically aligned Zn deposits (Supplementary Fig. 34a), with clear remnants of the LAPONITE coating (Supplementary Fig. 34b), and showed significantly lower O (28.9%) and S (3.1%) content (Supplementary Fig. 34d), indicating suppressed corrosion. The presence of Mg (1.6%) and its spatial localization (Supplementary Fig. 34c) confirm the retention of the LAPONITE layer.

On the cathode side, the MnO_2 from the LAPO@Zn-based pouch cell exhibited milder degradation, as indicated by fine flake-like $Zn_4SO_4(OH)_6 \cdot 4H_2O$ deposits (Supplementary Fig. 35b,c) and corresponding XRD signals (Supplementary Fig. 35a). In contrast, the cathode paired with bare Zn showed denser, sheet-like deposits (Supplementary Fig. 35d,e), consistent with the coexistence of $Zn_4SO_4(OH)_6 \cdot 4H_2O$ and

$Zn_xMnO(OH)_2$—an indicator of Mn dissolution. The more complex XRD patterns in the bare Zn group and the dual-phase byproducts suggest stronger pH fluctuations triggered by anode-induced side reactions.

These results indicate that LAPO@Zn effectively suppresses anode-driven corrosion and side reactions, which in turn reduces cathode degradation. Comparing the MnO_2 cathode and LAPO@Zn anode extracted from the same pouch cell, the cathode exhibited more pronounced degradation, suggesting that performance fading in the LAPO@Zn||MnO_2 pouch cell is predominantly cathode-limited under current conditions.

Supplementary Table 1. Zeta potential of the LAPONITE+CMC interlayer in pristine and post-deposition states (Zn deposition at 1 mA cm^{-2} for 1 h).

Sample status	Measurement No.	Zeta Potential (mV)
	1	1.9764
Pristine	2	2.2340
	3	3.6298
	1	11.9061
After deposition	2	12.6010
	3	13.3090

The observed increase in zeta potential after Zn deposition, from an average of 2.61 mV in the pristine state to 12.61 mV after deposition, indicates charge redistribution at the interface. In this measurement, the “after deposition” sample refers to the LAPONITE+CMC interlayer that was peeled from the Zn electrode after 1 h of deposition at 1 mA cm^{-2}. This change is attributed to selective ion interactions, where Zn^{2+} ions accumulate near the outer surface and SO_4^{2-} ions remain confined within the LAPONITE interlayer. These results support the proposed role of LAPONITE in modulating the local ionic environment by facilitating Zn^{2+} transport and anchoring SO_4^{2-}, thereby suppressing Zn^{2+}-SO_4^{2-} contact ion pair formation and promoting uniform Zn^{2+} transport.

Supplementary Table 2. Summary of application of DRT peaks calculated for a Zn||Zn symmetric cell.

DRT Peak	Approximate time constant, τ	Assignment
P1	11.22-12.88 μs	Double-layer relaxation
P2	19.5-22.38 μs	Solid-electrolyte interphase
P3	0.14-0.16 ms	Positive electrode charge transfer
P4	2.19-3.6 ms	Positive electrode charge transfer
P5	32.3-53.7 ms	Positive electrode charge transfer
P6	0.54-0.7 s	Diffusion
P7	5.75-6.1 s	

Supplementary Table 3. Concentration of elements in the electrolyte in LAPO@Zn symmetric cell systems from inductively coupled plasma (ICP) results.

	Mg (mg L^{-1})	Zn (mg L^{-1})
Before cycling	132.8	2,644,192.8
After 50 cycles	1,725.7	2,573,247.7

The Mg element concentration in the electrolyte (from dissolved LAPONITE) after cycling increases slightly, indicating the LAPONITE can be stably presented in the system during cycling.

Supplementary Table 4. Comparison of performance between previously reported pouch cells and LAPO@Zn||MnO_2 pouch cell.

Positive & negative materials	Electrolyte	Mass loading (mg cm^{-2})	Voltage range (V vs. Zn^{2+}/Zn)	Current density (A g^{-1})	N/P ratio	Cycle No.	Initial & final capacity (Ah)	Ref.
α-MnO_2 & Bi@Zn powder	2 M $ZnSO_4$ + 0.1 M $MnSO_4$	2	1.0-1.8	0.5	6.65	260	0.00515 & 0.00634	1
$V_2O_5 \cdot nH_2O$ & Zn foil	3 M $ZnSO_4$ + 80 mM 1-butyl-3-methylimidazolium cation	12.3	0.3-1.6	0.116	4.91	70	0.058 & 0.063	2
$ZnV_6O_{16}\cdot 8H_2O$ & Zn foil	2 M $ZnSO_4$ + 0.1 M N-Acetyl-ε-caprolactam	17.6	0.25-1.5	0.474	3.80	100	0.0375 & 0.034	3
α-MnO_2 & Electrodeposited Zn on carbon paper	2 M $ZnSO_4$ + 0.1 M $MnSO_4$	10	0.8-1.8	0.031	1.35	70	1.54 & 1.22	4
α-MnO_2 & Hydroxyapatite@Zn foil	2 M $ZnSO_4$ + 0.2 M $MnSO_4$	10	0.9-1.9	1.5	1.90	250	0.022 & 0.015	5
**γ-MnO_2 & LAPONITE@Zn foil**	**2 M $ZnSO_4$**	**8.94**	**0.8-1.9**	**0.1**	**3.57**	**105**	**1.967 & 2.753**	**This work**

Supplementary Notes

Supplementary Note 1. Additional properties of the LAPONITE+CMC coating layer.

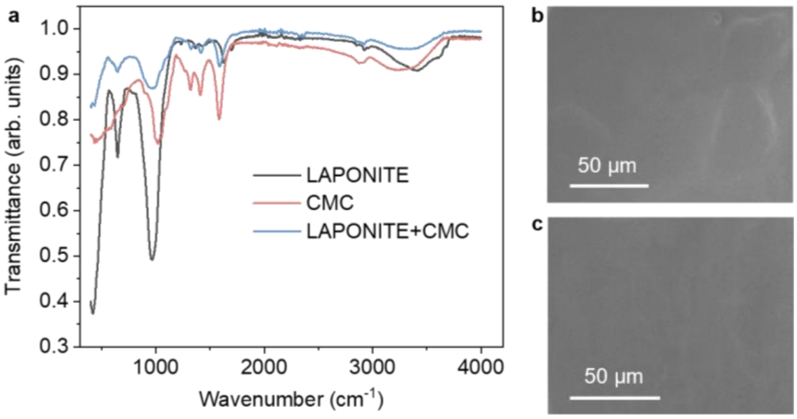

Supplementary Figure 36. Additional investigations of LAPO@Zn. a, Fourier transform infrared (FTIR) of LAPONITE, CMC, and assembled coating composed of LAPONITE and CMC (LAPONITE+CMC). The peaks of LAPONITE+CMC are similar to those of the initial LAPONITE and CMC, indicating that our cell fabrication process does not significantly change the nature of LAPONITE. **b,c,** SEM images of the LAPONITE+CMC. The morphology of LAPONITE+CMC is presented differently from Fig. 3a,b in the main text, eliminating the possibility that the cubes are from LAPONITE or CMC.

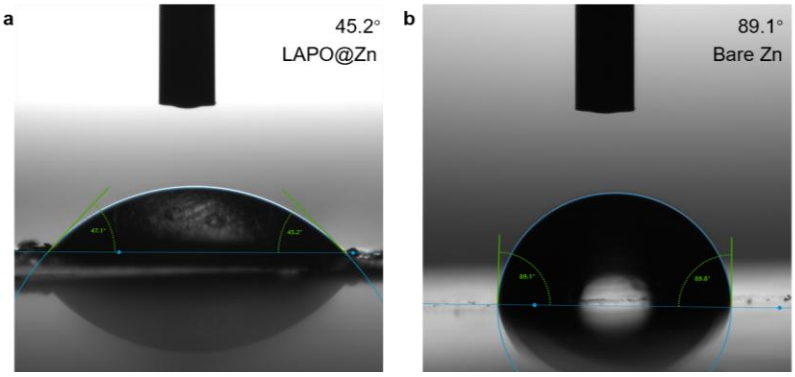

Supplementary Figure 37. Contact angle test on bare Zn and LAPO@Zn anodes. The contact angle of water on the LAPO@Zn surface is 45.2°, which is much smaller than that of bare Zn surface (89.1°), suggesting that LAPONITE interlayer favors electrolyte penetration and may contribute to Zn^{2+} diffusion.

Supplementary Note 2. physical meaning of the parameters used in CA analysis.

The parameters used in the CA analysis are defined as follows:

$$a=\frac{2\cdot\pi\cdot n\cdot F\cdot M\cdot h\cdot A\cdot N_0\cdot k_1^2}{\rho} \quad (3)$$

$$b=\frac{\pi\cdot M^2\cdot N_0\cdot k_1^2}{\rho^2} \quad (4)$$

$$c=n\cdot F\cdot A\cdot k_3 \quad (5)$$

$$d=\frac{\pi\cdot M^2\cdot N_0\cdot k_2^2}{\rho^2} \quad (6)$$

$$I_0=\frac{\Delta E}{R} \quad (7)$$

$$\tau_{\mathrm{DL}}=R\cdot C \quad (8)$$

Definition of symbols:

N_0 — number density of 2D or 3D active sites formed instantaneously;

k_1 — rate constant of the 2D growth of the nucleus;

k_2 — rate constant of the 3D parallel (lateral) growth;

k_3 — rate constant of the 3D perpendicular (outward) growth;

A — electrode surface area active for 2D or 3D nucleation and growth;

h — height of the 2D layer;

ρ — density of the deposited 2D layer or 3D layer;

M — molar mass;

n — number of electrons transferred in the deposition reaction;

ΔE — direct current potential step applied to the electrode;

R — solution resistance (equivalent series resistance);

C — double layer capacitance (equivalent series capacitance);

Supplementary Note 3. Effect of thickness of the LAPONITE+CMC coating.

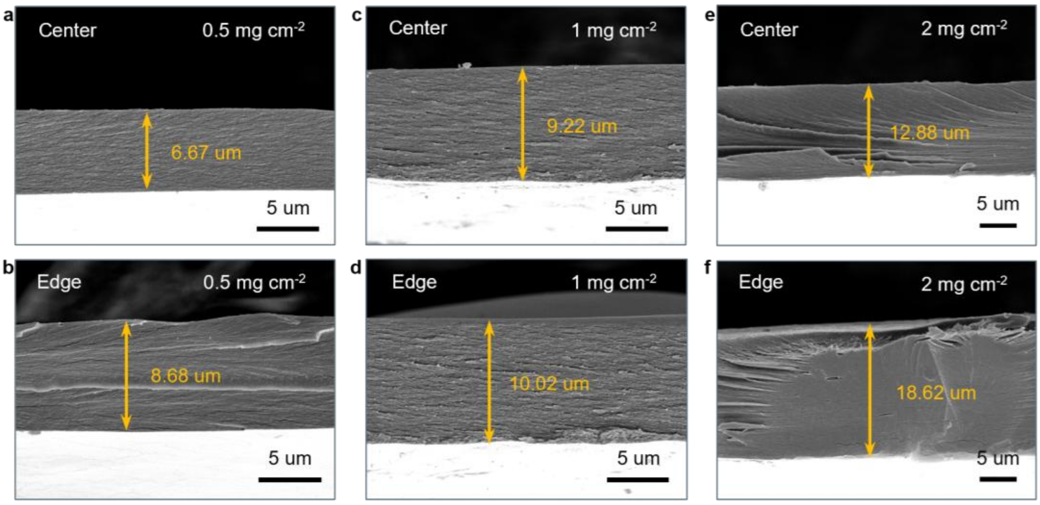

Supplementary Figure 38. Cross-sectional SEM images of LAPONITE+CMC coatings with different LAPONITE mass loading of (**a,b**) 0.5 mg cm^{-2}, (**c,d**) 1 mg cm^{-2}, and (**e,f**) 2 mg cm^{-2}. SEM images collected from the center region are shown in **a**,**c**,**e**, and those from the edge region are shown in **b**,**d**,**f**.

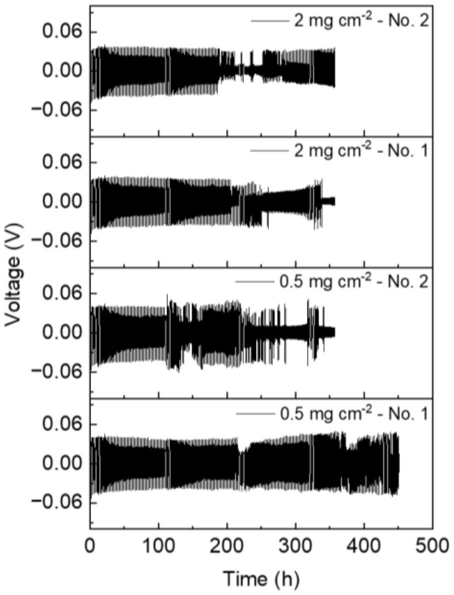

Supplementary Figure 39. Galvanostatic cycling curves of LAPO@Zn||LAPO@Zn symmetric cells at 1 mA cm^{-2} with a fixed capacity of 1 mAh cm^{-2}. The mass loadings of the LAPONITE are 0.5 mg cm^{-2} and 2 mg cm^{-2}, respectively. Each sample was tested twice to confirm reproducibility.

Cross-sectional SEM image (Supplementary Fig. 38) reveals that the 1 mg cm^{-2} LAPONITE+CMC interlayer exhibits superior structural uniformity, with a center

thickness of 9.22 μm and an edge thickness of 10.02 μm (8.78% variation). Vertically aligned gaps are observed across the coating layer, which may facilitate directional ion transport and effective Zn^{2+}/SO_4^{2-} separation. In contrast, the 0.5 mg cm^{-2} coating displays a 30.13% thickness variation between center (6.67 μm) and edge (8.68 μm), along with edge curling and microcracks. The 2 mg cm^{-2} coating shows greater variation of 44.57% (12.88 μm at the center vs. 18.62 μm at the edge), and widespread wrinkling and cracking. These structural inconsistencies are indicative of poor coating layer formation and compromised mechanical robustness. Correspondingly, the 1 mg cm^{-2} interlayer enables the most stable cycling performance, maintaining over 1,200 h in Zn||Zn symmetric cells (Fig. 5a in the main text), whereas the other two configurations fail before 500 h (Supplementary Fig. 39).

Supplementary Note 4. Reproducibility of the Zn||Zn symmetric and Zn||MnO_2 coin cells.

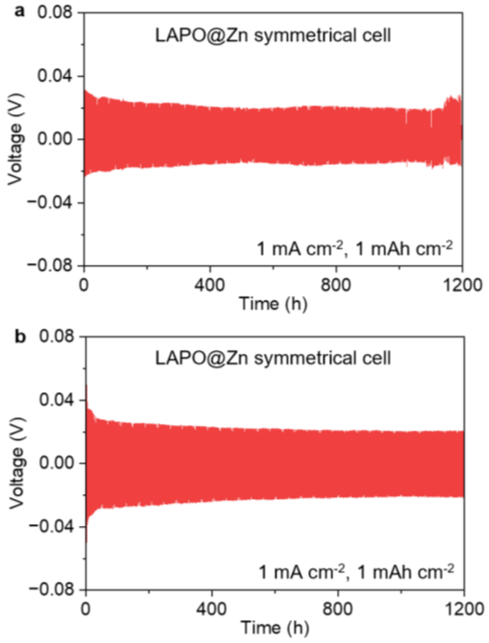

Supplementary Figure 40. Extended galvanostatic cycling profiles (complementing Fig. 5a in the main text) of symmetric coin cells with LAPO@Zn electrodes, tested at a 1 mA cm^{-2} and a fixed capacity of 1 mAh cm^{-2}.

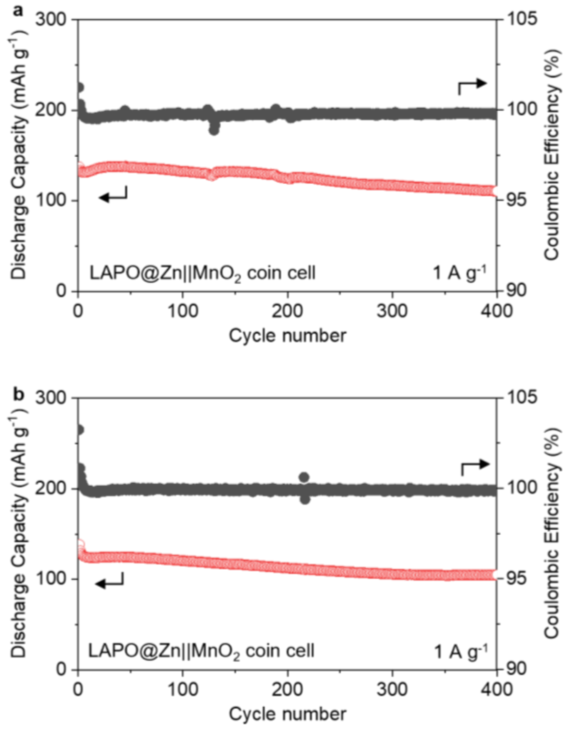

Supplementary Figure 41. Extended galvanostatic cycling profiles (complementing Fig. 5b in the main text) of LAPO@Zn||MnO_2 coin cells at 1 A g^{-1}.

Supplementary Note 5. Voltage profiles of Zn||MnO2 coin and pouch cells.

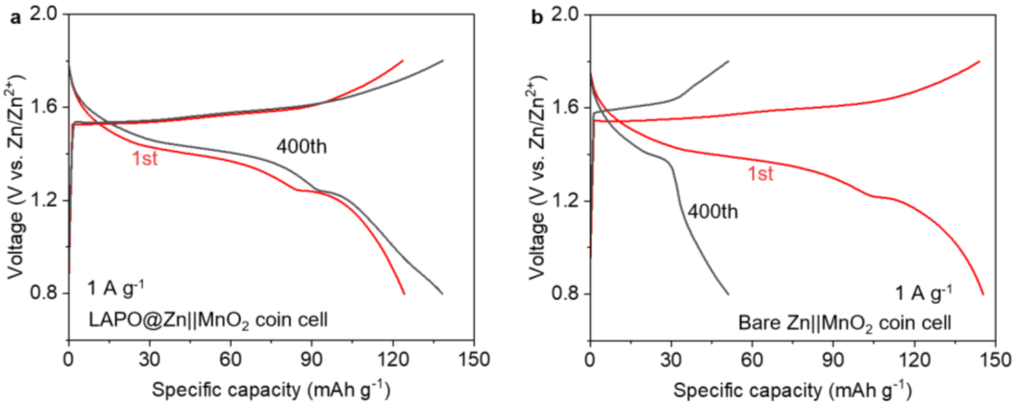

Supplementary Figure 42. Galvanostatic charge/discharge profiles of LAPO@Zn||MnO2 and bare Zn||MnO2 coin cells at different cycles, tested at 1 A g^{-1}.

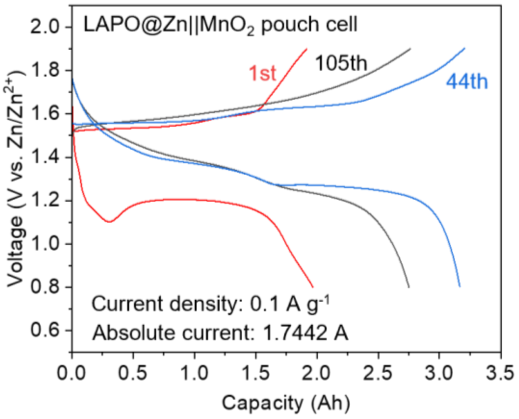

Supplementary Figure 43. Galvanostatic charge/discharge profiles of the LAPO@Zn||MnO2 pouch cell at different cycles, tested at a current density of 0.1 A g^{-1} and an absolute current of 1.7442 A.

Supplementary Note 6: CFD simulation method and parameters for Fig. 2.

The following equations and parameters were used in the simulations.

1. Governing Equations:

Navier-Stokes equations for incompressible fluid motion in the electrolyte (Ω_1).

Darcy's law for the porous LAPONITE coating (Ω_2), with Kozeny-Carman model-derived forces $Fporous = \frac{180\mu(1-\epsilon)^2}{dp^2\epsilon^3}u + \frac{\rho}{\epsilon^2}\nabla \cdot u$

Convection-diffusion equations for ion transport, incorporating free (c_{free}) and adsorbed (c_{abso}) ion concentrations.

2. Key Parameters:

- Porosity (ϵ): 1 (electrolyte) and 0.3 (porous LAPONITE layer).
- Nanopore diameter (dp): 1 nm.
- Diffusion coefficients: $D_1=0.8\times10^{-7}$ m^2 s^{-1} (electrolyte), anisotropic D_1 in the coating (enhanced along the channel axis), and $D_2\approx10^{-11}$ m^2 s^{-1} for adsorbed ions.
- Boundary conditions: Nonzero velocity at the top boundary to induce artificial vortices, zero velocity elsewhere; fixed ion concentration at the top (c_{free}=initial value), zero flux at side boundaries.
-

3. **Domain**: 2D region (100 × 100 μm^2) above the electrode.

Supplementary References

1. H. Chen *et al.*, Zinc iso-plating/stripping: toward a practical Zn powder anode with ultra-long life over 5600 h. *Energy Environ. Sci.* **17**, 3146-3156 (2024).
2. G. Ma *et al.*, Organic Cations Texture Zinc Metal Anodes for Deep Cycling Aqueous Zinc Batteries. *Adv. Mater.* **36**, e2408287 (2024).
3. D. Xu *et al.*, Chelating Additive Regulating Zn-Ion Solvation Chemistry for Highly Efficient Aqueous Zinc-Metal Battery. *Angew. Chem. Int. Ed.* **63**, e202402833 (2024).
4. H. Yang *et al.*, Sustainable high-energy aqueous zinc–manganese dioxide batteries enabled by stress-governed metal electrodeposition and fast zinc diffusivity. *Energy Environ. Sci.* **16**, 2133-2141 (2023).
5. Q. Zou, Z. Liang, W. Wang, D. Dong, Y.-C. Lu, A nuclei-rich strategy for highly reversible dendrite-free zinc metal anodes. *Energy Environ. Sci.* **16**, 6026-6034 (2023).
