# Supplementary material for: Mitigating ion flux vortex enables reversible zinc electrodeposition
Source: Nat Commun. 2025 Aug 8;16:7312. doi: 10.1038/s41467-025-62470-x (PMC12331982; doi:10.1038/s41467-025-62470-x)
Supplement: Supplementary file 2 — Description of Additional Supplementary Files [file 41467_2025_62470_MOESM2_ESM.pdf]

File Name: Supplementary Data 1
Description:
This plain text file (.txt) provides the atomic coordinates of the optimized computation al models used in the theoretical calculations.
